# Understanding intergenerational fitness tracking practices: 12 suggestions for design

**DOI:** 10.1007/s42486-021-00082-2

**Published:** 2021-11-16

**Authors:** Kavous Salehzadeh Niksirat, Fitra Rahmamuliani, Xiangshi Ren, Pearl Pu

**Affiliations:** 1 School of Computer and Communication Sciences, École Polytechnique Fédérale de Lausanne (EPFL), Lausanne, Switzerland; 2 School of Information, Kochi University of Technology, Kami, Japan; 3 Department of Information Systems, University of Lausanne, Lausanne, Switzerland

**Keywords:** Fitness tracker, Intergenerational, Older adults, Physical activity, Sedentary lifestyle, Design implications

## Abstract

The paper presents a qualitative study to explore the use of fitness trackers and their social functions in intergenerational settings. The study covered three phases of semi-structured interviews with older and younger adults during individual and intergenerational use of the fitness trackers. The study revealed comparability as common fitness practice for older adults. The findings show that intergenerational fitness tracking practices can increase in-person meetings and daily discourses and thus enhance family social bonds. An unexpected benefit of this practice is its ability to help older adults overcome technology barriers related to the use of fitness trackers. Overall speaking, families whose intergenerational members already enjoy a strong relationship are likely to gain the most from such practices. Many challenges remain especially concerning the motivation and involvement of younger partners and the user experience design aspect of such digital programs. For this purpose, we have developed some recommendations for the future development and deployment of intergenerational fitness tracking systems to stimulate interactions between younger and older family members and thus to promote their physical and emotional well-being.

## Introduction

The world population is aging. An aging society presents both opportunities and challenges. The difference crucially depends on the health conditions of older citizens. If they can live the extra years of life in good health and in supportive social environments, it is a blessing for them, and can have a positive impact on their families, communities, and the society in general. On the other hand, if the additional years are dominated by declines in physical and mental health, hospital visits, isolation from the society, and the threat of ageism, the outlook is rather pessimistic. Unfortunately, little evidence can be found indicating older adults today are enjoying better health than their parents. In fact, they are perhaps more prone to a sedentary lifestyle (Marmot 2005; Harvey et al. 2013) and its associated risks such as cardiovascular disease (Taylor et al. 2004) and dementia (Laurin et al. 2001). Thus, it is crucial to identify effective practices to encourage fitness activities for seniors.

An important motivation for individuals to participate in fitness activities, regardless of their age, is social support (Bandura 1986; Duncan et al. 2005), where many studies confirmed that having an exercise companion effectively promotes physical activity (Trost et al. 2002; Rackow et al. 2015). On the other hand, given recent advances in personal informatics and wearable technologies, fitness trackers have become increasingly popular (Vogels 2020). Researchers studied different social strategies on how to motivate physical activity using fitness tracking technology (Cherubini et al. 2020; Sullivan and Lachman 2017; Consolvo et al. 2006; Lin et al. 2006; Ren et al. 2018), and how to design for social fitness activities (Gui et al. 2017; Chen and Pu 2014; Puussaar et al. 2017; Epstein et al. 2015). But, these studies usually have been done with *intragenerational* peers (i.e., young–young or old–old).

In this paper, we focus on *Intergenerational* interactions between old and young users, and we hypothesize that using fitness tracking technology in an intergenerational social context can be beneficial for older adults. However, it is unclear how older adults perceive intergenerational fitness practices, what are the common fitness and social practices to maintain active life, and how intergenerational support might impact older adults’ fitness behavior. At the same time, using a fitness tracker poses significant technological barriers for older adults (Preusse et al. 2017; Kononova et al. 2019)—both from the tracker device itself and its related smartphone app. So a relevant question is how intergenerational support can help older adults overcome technology barriers. In this paper, we investigate the following research questions:

- **RQ1**. What are the intergenerational fitness tracking practices and what factors do facilitate or inhibit such practices?
- **RQ2**. How do these practices affect the social interaction of older adults with their younger family members?

To address our research questions, we report an in-depth qualitative study. We consider six different case studies—six intergenerational pairs. We conduct a 4-week qualitative study interviewing participants of each pair before the study, after individual use of the fitness tracker, and after intergenerational use of the fitness tracker. Besides, we monitor participants’ step counts, their motivation for fitness activities, and the frequency of meetings with their partners.

Overall, our study shows older adults’ interaction with the fitness tracker and with the younger partners improved after the individual use of fitness trackers followed by intergenerational practice. We further found that intergenerational fitness practices encourage in-person meetings and daily discourses between the generations, and as a consequence, social bonds between family members are strengthened. This social interaction helps older adults overcome technology barriers and facilitates fitness practices. At the same time, we identified socio-technical challenges that older adults faced in intergenerational practices.

Our work serves as an initial step for a nuanced and comprehensive understanding of intergenerational interactions to encourage an active lifestyle using fitness trackers. Most importantly, our work contributes to design and provides implications informing innovation of intergenerational fitness tracking systems that can promote the physical, mental, and emotional well-being of older adults in families.

The rest of the article is structured as follows. Section 2 presents background information about the impact of the intergenerational practices. Section 3 summarizes the related work about technological solutions that have been provided. Section 4 explains the experimental design and the procedure of the study. Section 5 reports on the qualitative and quantitative results. Section 6 discusses the findings by revisiting the research questions and provides 12 implications for the future design of the intergenerational fitness tracking systems. Last, Sect. 7 discusses limitations and future work, and concludes the study.

## Background

In this section, we review the overall impacts of intergenerational programs. Considerable research efforts have shown the impact of intergenerational programs on reducing negative attitudes towards older adults and for breaking down age stereotypes (Powers et al. 2013; Harwood 2007; Hernandez and Gonzalez 2008). Intergenerational interaction can improve the moods (Newman et al. 1995), affect (Kessler and Staudinger 2007), self-esteem (Kessler and Staudinger 2007), and life satisfaction of older adults (Kessler and Staudinger 2007; Powers et al. 2013). Past studies also identified the benefits of intergenerational interactions on strengthening family bonds (Bengtson 2001) and their potential impacts in reducing loneliness in industrialized societies (Vanderbeck 2007).

Familial intergenerational interactions also promote sharing health information between older and younger adults (Sandbulte et al. 2019). Furthermore, such familial interactions can facilitate digital literacy (Uhlenberg 2000) and contribute to learning *new* technologies for older adults (Mori and Harada 2010; D’Haeseleer et al. 2019; Leung et al. 2012). Older adults consider younger adults as more knowledgeable than other people in their generation and rather prefer to seek help from younger adults (Leung et al. 2012). Older adults can be highly dependent on their children and grandchildren to overcome the technology barriers (D’Haeseleer et al. 2019). One past work (Mori and Harada 2010) showed Japanese older adults who are living with larger families, for example, their grandchildren, learn how to use mobile phones more quickly than those who live with smaller families. Their findings also showed that older adults from larger families can better learn how to use advanced technology features such as taking photos and sending emails (Mori and Harada 2010).

## Related work

We review the prior work relative to technologies for intergenerational programs and fitness practices.

### Social support for physical activity

Social Cognitive Theory (Bandura 1986) provides a better understanding of how and why social support influences human behavior including physical activity. The validity of this theory has been attested to by many studies (Ahtinen et al. 2009; Booth et al. 2000; Leslie et al. 1999; Ståhl et al. 2001; Trost et al. 2002; Rackow et al. 2015). Being connected with family members and loved ones can motivate users to participate in wellness activities (Ahtinen et al. 2009). Earlier studies (Leslie et al. 1999; Ståhl et al. 2001) showed people who get less social support from families and friends had higher sedentary behaviors. Social support from peers was also found to be the main predictor of physical activity increments among older adults (Booth et al. 2000). Further, older adults who feel supported by their family and friends are more likely to be active than those who do not (Trost et al. 2002). Interestingly, this effect is higher if the partner is emotionally supportive (Rackow et al. 2015).

Prior work (Chen and Pu 2014; Lin et al. 2006) on fitness tracking practices investigated the dynamics of social support applying different social engagement strategies such as competition, collaboration, and cooperation. Previous studies (Gui et al. 2017; Lee and Lim 2015) have shown how fitness practices in online social networks can impact users’ social interactions and their fitness practices. Last, Walden and Sell (2017) explored the social dimensions of Fitbit fitness trackers, showing that pairing older adults together in peer groups can create positive peer pressure and promote physical activity. Although these studies addressed social support in fitness trackers, they mainly examined “intragenerational” peers (i.e., young–young or old–old).

### Older adults and fitness trackers

Advances in health informatics facilitate access to wearable devices for older adults. Earlier evidence (O’brien et al. 2015; Randriambelonoro et al. 2017; Sullivan and Lachman 2017) showed fitness trackers are promising tools to contribute to the health of older adults. Despite these benefits, fitness tracker abandonment is yet an issue. Older adults may stop using fitness trackers after short-term use. A previous work (Fausset et al. 2013), examining older adults’ attitudes in using fitness trackers, showed while older adults had a positive mind in the beginning, some of them changed their opinion after long-term use and abandoned it. Kononova et al. (2019) revealed that trouble with trackers is the main barrier for older users to maintain fitness tracking practices. Preusse et al. (2017) identified different usability issues for earlier models of fitness trackers (e.g., difficulty to navigate), and proposed creating video tutorials to facilitate the learning of difficult features for older adults. Given these usability barriers, using fitness trackers in intergenerational settings might be promising where younger family members could support their older counterparts to overcome the technical deficiencies.

### Intergenerational fitness practices

A recent review revealed intergenerational interactions are a promising approach to promoting active lifestyles (Flora and Faulkner 2007). For instance, engaging three generations of women (grandmother, mother, daughter) in a 6-month randomized controlled trial showed improvement in the level of activeness (Ransdell et al. 2005). These studies, however, paired the participants in experimental setups without using fitness trackers. Some researchers deployed emerging technologies for intergenerational users focusing on motion video games (Rice et al. 2013; Khoo et al. 2008). These technologies were even used in elderly care houses in the form of intergenerational programs, e.g., the Konnectics program used Microsoft Kinect (Matthew 2013). LIFE^1^ is another intergenerational program where health care practitioners from the younger generation trained and accompanied older adults for gameplay. Although these programs encourage physical activity, their user population is limited to special groups (e.g., frail or institutionalized older adults). In addition, playing video games is an occasional activity for many older adults, thus its benefits might be limited.

Family members share different types of health information (Binda et al. 2018; Grimes et al. 2009; Sandbulte et al. 2019; Li et al. 2020; Sandbulte et al. 2020). Saksono et al. (2020) designed a mobile app for young parents and their children to encourage sharing fitness information in families with low socioeconomic status. Their study revealed that parents were mainly satisfied with the app if they feel connected to their children and if they feel they can monitor and support their children using the app (Saksono et al. 2020). More related to our study, Li et al. (2020) studied how intergenerational family members might experience fitness data sharing through an online social network. This study asked participants to chat and share their fitness data in a chat group using WeRun.^2^ The study showed while older adults have more technical concerns with the use of the social network, the younger participants had more privacy concerns to avoid being tracked by their families. Where this study focused on online social network platforms, our work aims at understanding everyday practices that are mostly carried using bracelets and smartphone apps.

To conclude, it remains to be established what older adults’ attitudes are towards fitness tracking devices when receiving support from their family, what the typical intergenerational practices are, and how they influence older adults’ fitness activities.

## Method

Since intergenerational interactions are reciprocal social activities, we conducted our study with both older and younger generations. We mainly focused on the contextual understanding of older adult behaviors as well as younger adults’ roles in intergenerational interactions.

Intergenerational interactions could have different types where older adults may engage with younger adults among their family (Bengtson 2001; Mori and Harada 2010; D’Haeseleer et al. 2019; Thornton et al. 1984; Fingerman et al. 2012; Brigit et al. 1997; Sandbulte et al. 2019; Li et al. 2020), neighbors (Saksono et al. 2018), colleagues (Seymour et al. 1993), or with strangers for the sake of voluntary social activities (Kessler and Staudinger 2007; Newman et al. 1995; Powers et al. 2013; Hernandez and Gonzalez 2008; Tan et al. 2006). However, in this study, we will only focus on “family-based” relationships as it is the most common type of intergenerational relationships.

**Fig. 1:**
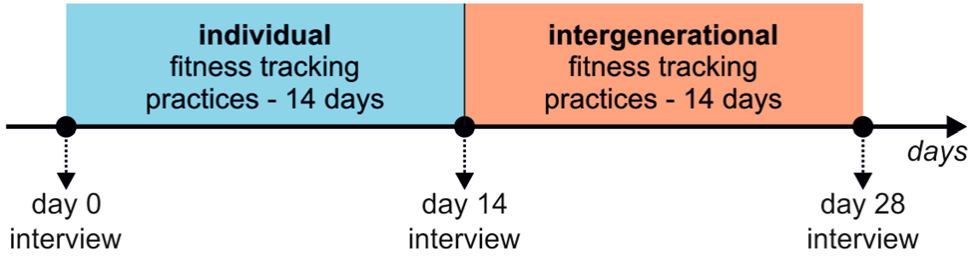
Timeline of the study design

Following previous studies (Knaving et al. 2015; Patel and O’Kane 2015; Gorm and Shklovski 2016; Preusse et al. 2017) that show an average of four weeks is a suitable time span for similar types of research, we ran a 4-week long qualitative experiment. Figure 1 shows the timeline of the experiment. Given that older adults require some time to learn how to use fitness trackers, the experiment included two weeks of using the tracker *individually* and two weeks of using the tracker in an *intergenerational* setting. A total of three semi-structured interview sessions were conducted: before the study, after the individual use, and after the intergenerational use.

### Apparatus

Fitbit Charge 2 together with Fitbit mobile app were used for fitness tracking. We used Fitbit Charge 2 since it has recently been used in several studies with older adults (Tedesco et al. 2019; Collins et al. 2019). Fitbit app is a well-known mobile app with over 28 million active users.^3^ To avoid further usability problems with smartphones, we asked participants to use their personal smartphone for running the app. ATLAS.ti software was used for analyzing qualitative data.

### Participants

We recruited participants through the snowball sampling method (Biernacki and Waldorf 1981), where the first pair was recruited through an in-campus advertisement and each pair introduced us to the next *candidate* pair to be recruited. We did our best effort to maintain a good diversity in terms of relationships between intergenerational partners where we included partners with different types of relationships: nuclear family through blood (e.g., father and son), nuclear family through marriage (e.g., father-in-law and daughter-in-law), and extended family (e.g., aunt and cousin). We also applied several additional selection criteria during participant recruitment: (i) Older participants should be at least 65 years old; (ii) Younger participants should be at least 18 years old; (iii) To control the factor of physical distance between partners, we recruited only those persons who lived not farther than 30 km from each other; (iv) To control the tech expertise issue, we only recruited participants who had experience using smartphones or tablets for more than 1 year and did not have prior experience using fitness apps *in intergenerational settings*; (v) Participants should have access to the Internet which is required for syncing the fitness trackers; (vi) Older participants should obtain a score of 25 or more in the Mini-Mental State Examination (MMSE) (Folstein et al. 1983). MMSE score of 25 or more indicates that older adults have the adequate cognitive capability to have an autonomous life (Crum et al. 1993).

After the selection process, we recruited 12 participants (six pairs) including six older adults (M = 68.8 years old, SD = 2.1, range = 65–71) and six younger adults (M = 39.3 years old, SD = 3.1, range = 33–41). While none of the younger participants had medical problems, older participants had different health issues including asthma (2/6), cardiovascular disease (2/6), sleep disorder (1/6), and leg pain (1/6). Despite these health issues, all participants were living independently. Detailed information about participants and their relationship with each other is provided in Table 1.

**Table 1:** Participants’ information. ‘O’ and ‘Y’ denote ‘old’ and ‘young’, respectively

ID	Relationship$^{\mathrm{a}}$	Sex	Age	Job	Children	Grandch.$^{\mathrm{b}}$	Living$^{\mathrm{c}}$
O1	Mother-in-law	Female	70	Housewife	3	5	Together
Y1	Daughter-in-law	Female	41	Clerk	2	0	Together
O2	Father	Male	71	Retired	2	4	Separate
Y2	Daughter	Female	41	Housewife	2	0	Separate
O3	Father	Male	65	Agency officer	2	0	Separate
Y3	Son	Male	33	Clerk	0	0	Separate
O4	Aunt	Female	69	Care helper	2	2	Separate
Y4	Nephew	Male	40	Company worker	2	0	Separate
O5	Father	Male	70	Retired	2	1	Separate
Y5	Daughter	Female	41	Company worker	1	0	Separate
O6	Mother	Female	68	Care helper	3	6	Separate
Y6	Son	Male	40	Interior designer	1	0	Separate

$^{\mathrm{a}}$Except for Y5, all participants were married

$^{\mathrm{b}}$Shows number of grandchildren

$^{\mathrm{c}}$Shows living conditions if the peers live together or they live separately

### Metrics

We collected qualitative data as our primary metric through three stages of interviews. As a quantitative metric, we collected participants’ step counts measured by the fitness trackers. Fitbit provides 1440 step count measurements per day, where we computed the averages of total daily steps before and after intergenerational uses. We also asked two structured questions (on a Likert scale from 1 to 5) about (i) the level of motivation they had for fitness activity in the last two weeks, (ii) the frequency of communication with their partner in the last two weeks. Last, we assessed perceived social self-efficacy using the self-efficacy scale questionnaire that measures one’s confidence to engage in social activities (Sherer et al. 1982; Smith and Betz 2000). The social self-efficacy subscale includes six items (on a Likert scale from 1 to 5).

### Task and procedure

After being informed about the experimental procedure, participants signed consent forms. Demographic information was collected. Next, participants attended a brief interview session. The initial interview was conducted in order to collect information about participants’ background including everyday life, social life, physical activity routines, health, technology proficiency, and experience with fitness trackers. Right after the initial interview, participants received a fitness tracker. A tutorial was given to the participants explaining how to use and wear the tracker, how to synchronize the tracker with the app, and how to track fitness information using the mobile app. Participants were asked to refrain from using any other fitness app and trackers during the whole experiment.

We considered the first two weeks of the experiment as a warm-up period where it provides enough time for older participants to practice wearing trackers and using the apps. In addition, we aimed to understand participants’ behaviors and attitudes while using the fitness tracker individually. Even though participants were recruited as pairs, we *explicitly* asked the younger partners to *refrain* from intervening on behalf of their older partners including either motivating or demotivating them for physical activity. Also, the app interface did *not* allow users to share performances and to exchange messages. At the end of the second week, we conducted the second interview, where we examined user attitudes, requirements, and concerns regarding the two weeks of fitness tracking experience. We asked what kind of strategies they used to increase their activeness, how they interacted with the tracker, and how motivated they were for fitness practices. After the interview, we asked the two structured questions mentioned earlier (cf. Sect. 4.3).

Right after the second interview (on the same day), we paired participants with their intergenerational partners. To this end, we used Community; a built-in social function of the Fitbit app. Community allows users to be paired and interact with other users by sharing their achievements, sending likes (e.g., cheers/taunt emojis), or chatting with each other on the Fitbit communication panel. The Community function can be also used with a group of users (i.e., more than two people), but given the focus of this study, we paired participants only with one partner. Participants should use the fitness tracker in tandem with their partners so that they could see each other’s performance and interact through the app. At the end of the experiment, participants were asked to attend the third interview. The setup of the third interview was similar to that of the second interview where we were interested to understand users’ behaviors in intergenerational settings and the relationship between physical activity and social connectedness. We also repeated the two structured questions used in the second interview (i.e., regarding the level of motivation and the frequency of communications). We first interviewed the older partner and then the younger partner. All the interviews were conducted in the university lab, but the interviews with the fifth pair were conducted in a local coffee shop. All interviews were video recorded for further evaluation. At the end of the experiment, we allowed participants to keep the fitness trackers as compensation for their participation.

### Rationale for selecting the study location

Japan has the highest rate of the elderly population, where over 28% of the people are over 65 years old (PRB 2020). The rural area of Japan presents many opportunities for our study. Health care of the elderly in rural areas is a more significant issue than in urban areas, where a greater proportion of people living in rural areas of Japan are elderly. For example, the Kochi prefecture where the experiment was conducted has around $33\%$ older population (Tokyo has $23\%$). It also ranks highest for outpatients per day amongst all prefectures in Japan. Thus, to solicit feedback from users in areas with the most risk factors, we decided to conduct this study in a rural area of Japan. On the other hand, based on a previous study (Hashizume et al. 2009), older adults living in rural areas have less tendency to use new technologies compared with those living in urban areas. This finding is consistent with current statistics which shows that the smartphone usage rate for older adults in rural areas is low ($44\%$ in the Kochi prefecture). However, the latest trends show the new generation of older adults will use smartphones much more in the upcoming years. For instance, in the Kochi prefecture, smartphone ownership has almost tripled over the last 7 years.^4^ Given that urban areas usually have higher smartphone coverage than rural areas, we think this work can be well extended to urban areas in Japan.

### Data analysis

We collected data from 36 interviews (12 participants $\times$ three interviews). With the help of a native Japanese speaker (fluent in English), we obtained the entire transcripts in English. To analyze the interview content, we used the thematic analysis method (Braun and Clarke 2006) focusing on both semantic and latent features of data. After familiarizing ourselves with data by iterative reading, we started the coding process in an inductive approach [i.e., analysis grounded in the data (Braun and Clarke 2020)]. We then generated an initial list of subthemes. Later, coauthors discussed the relevance of subthemes to merge, remove, or keep the subthemes for developing the main themes. We applied the methodology separately for two age groups and different interview phases. We later identified commonalities and differences between the age groups and the interview phases. Last, we selected relevant quotes from participants to report in the paper. We identified participants using the age groups and numbers (e.g., O3 is the older participant in the third pair; Y2 is the younger participant in the second pair).

## Main findings

To keep the article concise, we summarize the findings regarding the fitness practices during the individual use and focus on the main findings regarding intergenerational practices. We describe our findings on how participants perceived the intergenerational fitness practices, their attitudes, and expectations, and how such practices impact their social interactions. We highlight the effects of younger participants on both fitness and social practices and present underlying reasons why they might lose interest in intergenerational interactions. Last, we conclude the findings by presenting barriers to engagement and user expectations.

### Summary of fitness practices during the individual use

Our findings regarding the individual use of the trackers are inline with the existing literature where self-reflection (Randriambelonoro et al. 2017; Kononova et al. 2019), goal settings (Sullivan and Lachman 2017; Kononova et al. 2019; Preusse et al. 2017), and playfulness (Randriambelonoro et al. 2017) were the most well-received tracker features by the older adults.

Five out of six older users (henceforth 5/6) reported that self-reflection helped them increase their awareness about their activeness level. O6: “*I was always thinking if I am exercising enough. Now I am convinced that I am very active. I won’t have to worry.*” 2/6 older users mentioned using the tracker helped them learn their own activity patterns. O3: “*Using the tracker I learned my patterns of activeness such as my step numbers on working days, weekends, and the days I played golf.*” One of the older users mentioned that using the tracker supported self-reflection practices by facilitating how he was organizing his data before. O5: “*I had to note down how much I ran when I used a ‘Manpo-Kei’.*^5^*Now I have all my records [on the tracker].*”

Goal setting was the main strategy for older users’ fitness practices. O3: “*Usually, in the morning before taking the dog for a walk, I check the weather forecast and my body conditions. Then I set my goal. If all good, I set for more steps [goals].*” O5: “*I set* 10,000 *steps as a baseline goal to achieve. If I pass that baseline, I set a higher goal, maybe* 20,000.” While achieving daily goals was rewarding for older users, not achieving them caused a feeling of guilt. O5: “*When I missed daily goals, I felt I would be scolded by the tracker.*” Half of the older users described their experience with the tracker as playful. O1: “*I walked to a convenience store one day. In front of the shop, I was waiting for the firework to launch, and there it went off! I said, ‘Wow!’ *” The firework is a metaphor that Fitbit fitness tracker uses to gamify and promote daily goal achievements. 4/6 older users expressed a feeling of accomplishment and pleasure when they saw they achieved the goal. O1: “*The tracker celebrated with me. I felt I did my best.*” Besides the firework, users also considered receiving badges as a motivating factor to keep themselves active. O5 could make a meaningful relationship with the received badges: “*I had a bit of a hangover after a night party. I can hardly move! But after receiving a helicopter badge when I reached the upstairs [bedroom], I became more motivated.*”

### Fitness practices in intergenerational settings

#### Comparison stimulates activation

After getting paired with younger partners, older users reflected on their partners’ data. 5/6 older users compared themselves with their younger partners. O3 reported competing with his partner: “*Since we were connected [through the app], we naturally started competing. You know, I didn’t want to lose to my son.*” Y3 confirmed his father thoughts: “*Once I sent him a message that I moved up and down the stairs twice, then he suddenly replied that he did it six times!*” Y3 also reported an increase in his motivation for physical activity after seeing his father’s activity: “*We never before had an opportunity to check each other’s number of steps. So, I think it became a source of motivation for me to exercise more.*”

**Fig. 2:**
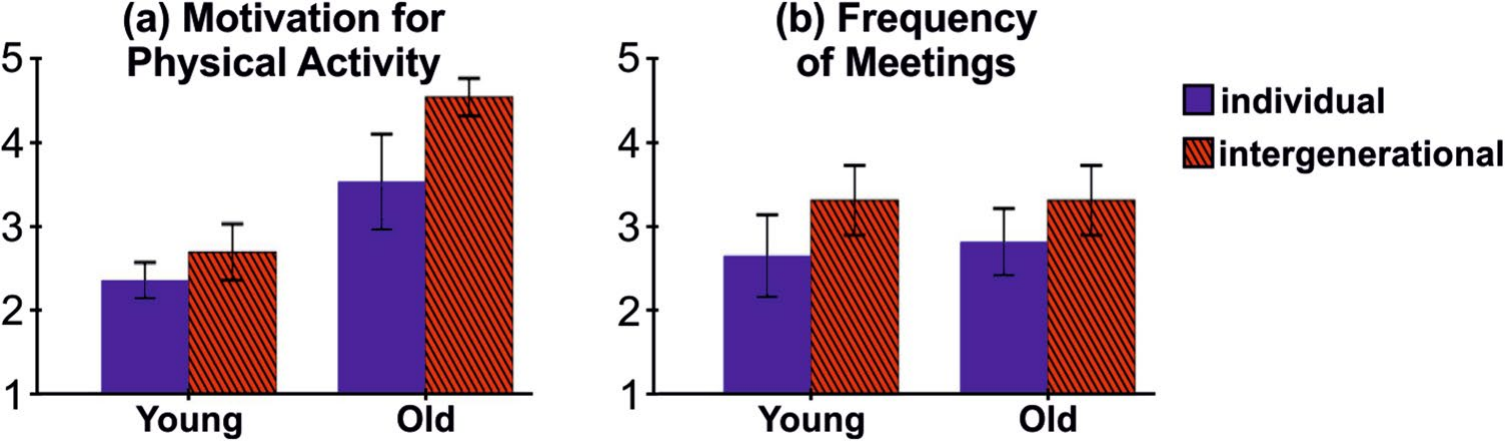
The quantitative results show: (**a)** how users in different age groups were motivated to exercise in the individual (dark blue) and intergenerational (hashed red) settings (1 = *not at all motivated*, 5 = *extremely motivated*), (**b)** how often users meet in-person during the experiment (1 = *never*, 5 =*very often*). The error bars indicate standard error

Reflection on one’s partners’ data increased the user’s motivation for physical activity. This finding was reflected in the quantitative question where *motivation* for physical activity was marginally increased (Wilcoxon signed-rank test: $Z = -1.897$, $p = 0.058$). This finding was more evident for older users than younger ones (Fig. 2a). Furthermore, motivation increase was partially reflected in daily step counts, where we noted a trend toward increase (though not significant) in the step counts of all participants (Wilcoxon signed-rank test: $Z = -1.334$, $p = 0.184$). This mild trend was more evident for older users, where 5 out of 6 older users took more steps during the intergenerational use than the individual use (Table 2).^6^ O6 was the only older user who has a lower step counts during the intergenerational use. This might be because she was already a very active senior person with over 14,000 average daily steps.

**Table 2:** Step counts during the individual and intergenerational uses of the fitness trackers

Participant ID	Individual	Intergenerational
O1	6701	7197
O2	5897	6352
O3	12,684	13,572
O4	9100	9660
O5	9228	9529
O6	14,836	14,157
Y1	4829	5718
Y2	6013	4803
Y3	9140	10,476
Y4	13,551	13,467
Y5	5388	5348
Y6	7840	8011

#### Partners’ validation

Several participants actively interacted with each other to validate their partners’ activeness. O1: “*I sent her messages saying ‘You’ll achieve with a little bit more effort.’ She also complimented me like saying ‘You’re doing well mother!’ *” Y1 confirmed her mother-in-law’s comments: “*She always sent me applause, cheers, or messages. I got many messages showing she cared about me.*” Y3: “*I was losing to my dad so it didn’t make sense to send him a ‘taunt’. I usually sent him messages informing my conditions, for example, ‘I just finished jogging!’ *” O6: “*I had more steps than my son, so I thought it is not good to send him a ‘taunt’. Instead, I sent the ‘cheer’ and messages like ‘I’m trying my best, do your best too.’ *”

O1 described interacting with her partner only if she thought her partner needed support: “*Sometimes she was over* 5,000 *steps. So, I didn’t feel that I needed to send another message. She was busy during the day. I didn’t want to disturb her.*” According to O1’s comment, she considered 5000 steps to be enough for her partner, indicating that the partner validation is a very subjective judgment. In addition, while validating each other’s fitness data, most of the older users (5/6) perceived their own achievements differently compared to those of their partners. O1: “*Our data is not similar. Her data was obtained only from doing her work, but mine is different as I go for a walk to achieve the goal.*” O3: “*I was thinking why the older father should have more steps than the younger son. Probably because our life, the place we live, and our working styles are different.*”

#### Older adults performed beyond expectations

Our older participants walked on average 9909 steps per day (median = 9378), which is meeting the criterion for an active person [i.e., 7100 to 11,000 steps per day (Tudor-Locke et al. 2011)]. More interestingly, 5/6 older users logged more step counts than their younger counterparts. This was reflected in the interviews with younger users, where they (4/6) expressed the feeling of wonder after reflecting on older users’ physical activity. Y1: “*It was awesome! She was doing more exercise than I did. I envied her!*” Y2: “*I was surprised when I saw my father’s calorie consumption. He was moving too much.*” Y5: “*I had always imagined my father was sitting the whole day at home. When I saw he walked more than me, I wondered where he was walking to get these many steps.*”

Beside physical activeness, some of the older adults also behaved unexpectedly in terms of a curious use of the tracker. 3/6 younger users mentioned how they were surprised to see that their older partner was proactively using the tracker. Y1: “*My mother-in-law was more interested in the tracker than I was. She explored different features of the tracker and showed me the calorie, heart rate, and sleep recording functions.*” Y6: “*My mother thought I didn’t use the app properly. So, she took screenshots of different functions and sent them to me. She was trying to teach me!*”

### Intergenerational fitness practices increase social interactions

#### Intergenerational fitness practices re-attach family members

Users perceived intergenerational fitness practices to be beneficial to their social interactions. Some users believed these practices improved their social bond with their partner, becoming more thoughtful about their partner and better understanding them. Y1: “*I had never thought about how my mother spent her days until now. After using it [the tracker], I started wondering. I also felt somewhat responsible for her. I had a feeling that I saw a different side of her. I had a chance to think more about her, for example, what she is doing now and whether she can walk today because of the heavy rain.*” O6: “*When my son was young, I was caring much about his life but later I stopped. Using the tracker, I became aware of his conditions again!*”

In addition, half of the users from both generations reported tracking the activity of their partner for the sake of health care. Y2: “*He is getting old. If he wears it all the time, I can monitor his conditions and deal with emergency situations, for example, if his movement stops* $\dots$” O2 echoed his daughter: “*It is very useful if my daughter knows how I move because there is a lot of distance between my house and her house (25 km). When I get older, she’ll know my condition.*” Half of the younger users expressed their cheerful feeling after becoming aware of their partner’s activeness. Y1: “*I felt happy when I saw my mother reaching 10,000 steps on some days. I was glad that she was fine on those days.*” Y3: “*I want my father to lose his weight, so I think it was good to see he was walking a lot! It was meaningful to invite him to this experiment.*”

Last, 3/6 older users reflected on younger partner data to infer what they are doing. O6: “*Well, when I looked at my son’s data he was not walking much. After all, I understood that he had a routine day.*” O1 reflected on her daughter-in-law’s data to inform her 7-year-old grandson: “*Evenings is the time when my grandson gets bored and misses his mother. I showed my daughter-in-law’s activity data to him saying, ‘Boy, look at this! Your mom works hard, so, please wait a little bit more.’ *” O6 also described how she could speculate from her son’s reaction to her activeness: “*My son sent me a message saying my step numbers were amazing. But my step numbers were moderate, so I understood that he was not walking much (laughing).*”

#### Stimulate in-person meeting to face technological challenges

Our findings highlighted the importance of in-person interactions in intergenerational fitness practices. Older users perceived that in-person meetings were actually facilitated by the use of this technology, where these meetings supported older users to overcome technology barriers. For example, O1 had difficulty while inputting text in the smartphone and preferred to share information in-person: “*It is better to directly meet because it is difficult for me to type. I also got interrupted when I wrote something in the app. So I prefer talking and showing rather than just typing.*” Due to technology barriers, O2 preferred to sit together with his daughter and check the results in-person: “*We opened the app together, synced the* Fitbit, *then looked at our data, and talked about it.*” Y2 also added: “*When we met, I tried to teach him how to sync the app.*” O5 and Y5 also described how they practiced interacting with the technology during in-person meetings. O5: “*We looked at the data together when she came home at night.*” Y5: “*We practiced sending and sharing the data with each other when we were speaking in-person. Then, we could talk about the data.*” O1 and Y1 practiced fitness together in a social event since O1 believed it can increase their participation in general life activities. O1: “*We sometimes dance in our religious event. I learned that when I dance one hour during the whole event, I could achieve* 10,000 *steps. I did together with my daughter for half an hour, stepping back and forth at a distance of* one *meter. I could reach* 5,000 *steps. I told my daughter that ‘God’s dance is good for our health’ and we were laughing.*” Such findings were also reflected in the questionnaire data where the number of personal meetings between partners outside the fitness training increased during the intergenerational use (Wilcoxon signed-rank test: $Z = -2.646$, $p = 0.008$, Fig. 2b).

We also found that physical distance impacts the in-person meetings. Y1: “*Since we live together, besides interacting with the app, we also met and talked directly.*” Conversely, two older users alluded to the same principle by saying that they sometimes missed the opportunity to get support because they live far from their younger partners. O2: “*When I faced technical problems, I was not able to freely communicate with my daughter because she was not here.*” O4: “*I had a synchronization problem. I asked him for help. Unfortunately, it wasn’t possible because he couldn’t drop by.*”

#### Content changed in daily discourse

Although we obviously did not pair the participants in the first two weeks, the younger and older participants still had chances to exchange their thoughts. Nevertheless, the conversations during this time were limited to small talk about how to wear the tracker. For example, several participants discussed whether they remember wearing the tracker every day. Y2: “*I asked him ‘Did you put it on today?’ ‘Are you wearing it?’* ” After introducing the intergenerational settings, the discussion content notably changed to deeper conversations about how they practiced fitness and to the content and meaning of their fitness data. O1: “*We talked about the routes that my daughter walked every day. Her office is far from the elevator. So, if she takes the elevator, she needs to walk more. But if she takes the stairs then she’ll log more floors but fewer steps.*” Y1: “*We found it is different if you climb stairs in the house, in my workplace, or in the church. Our house stairs are not that high, and they didn’t count as one floor.*” O5 investigated his daughter’s sleep patterns to use it as a reference for himself. Y5: “*My father always reminded me to wear my tracker properly, otherwise my heart rate won’t be recorded precisely. He was expecting to see fluctuation in the data, but my sleep data was mostly flat at night. He thought I was not wearing it properly, but it is not true. It is because I slept well.*”

Participants also reported having technical discourse during both individual and intergenerational use of the trackers. Three pairs reported technical discussion. For example, O2, an older user that faced the most technical difficulties compared to other older users, reported: “*I usually, asked a lot of questions from my daughter about how can I use it. For example, I didn’t know how to sync the smartphone with the tracker. I gave my smartphone to her to solve the issue.*” His younger partner added, Y2: “*Rather than interacting through our steps, I spent a lot of time to train my father how to overcome the burden of use.*” Different older users mentioned various technical challenges such as O3: “*I got a connection problem* [$\dots$]”, or O4: “*I mistakenly uninstalled the app* [$\dots$]”.

### The younger partner’s role in the partnership: when young partners lose interest

The interviews showed younger users in half of the pairs took a more active role in terms of supporting technical issues and accompanying older users in daily discourse. Nevertheless, we also found some younger users who did not support their older partners enough for several reasons such as being busy with daily life, lack of interest, or lack of emotional bonds between them.

Half of the older users (O4, O5, O6) mentioned their younger partner was not interested enough to interact with them. For example, O4 said: “*In the beginning, I sent him some messages and shared with him my step counts, but he didn’t reply. I sent him a message to check if it is already delivered. It seems it did.*” O5, after seeing her daughter’s lack of interest, decided to use the tracker individually: “*The interaction between us didn’t go well. It seems she was not interested in how much I am walking. I thought it doesn’t make sense to share and I shall do this by myself.*” O6 reported that her son was using template messages to reply to her achievements making her wonder, receiving repetitive impressions: “*I sent him my data, but he rarely replied, ‘It’s great!’ (cynical). When he did, I didn’t know why I got identical messages three times in a row. Always the same message, ‘It’s great!’ (cynical). Nothing more.*”

Users spoke about different reasons why they could not build a fruitful relationship either to support fitness practices or through partnership in fitness practice. For example, one of the users believed because his daughter doesn’t share similar fitness goals with him, this could negatively affect the fitness practice. O5: “*Her life was routine without much effort for exercising. Her data was not something meaningful for me. If the family members have similar intentions to set their own fitness goals and cooperate towards those goals, then it might work.*” O5 also experienced some sort of family estrangement: “*I usually see my daughter every night when she picks up my grandchild, but even at that time we don’t have any conversation. There aren’t many common topics between us.*” O5 believed her daughter’s busy lifestyle was the main reason: “*She is very busy at work. I think that is why we don’t understand each other well.*”

**Fig. 3:**
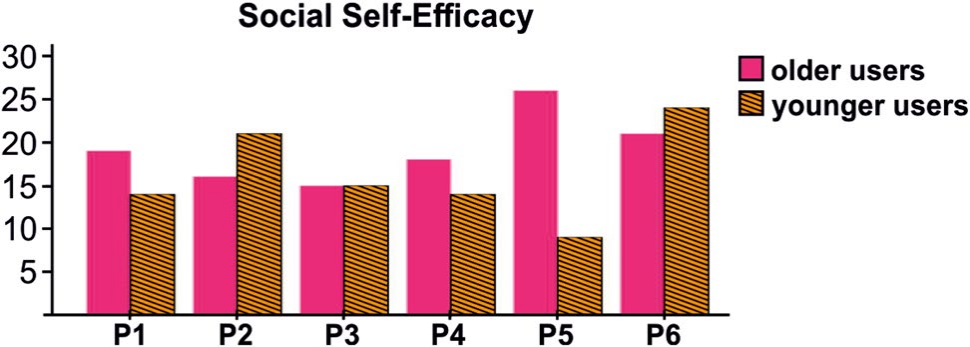
Perceived social self-efficacy. The vertical axis shows the total of the six items (for each item: 1 = *strongly disagree*, 5 = *strongly agree*). The horizontal axis shows each pair (P). The older and younger users were shown with pink and hashed orange, respectively. The results show Y5 had the lowest score amongst all users

While O5 thought being busy was the main reason for his daughter’s lack of interest, the daughter believed using technology together with a buddy was not an interesting idea. Y5: “*It is nice that my father walks a lot, but I am not so much interested in these kinds of relationships. I prefer to get encouraged by technology rather than my family members! If the people tell me that I should exercise, I will say, ‘Leave me alone please!’ *” Y5’s thoughts were in line with the results of the self-efficacy questionnaire where she had the lowest *perceived social self-efficacy* among all participants (Fig. 3) potentially indicating her low confidence to join a social fitness program and to initiate an intergenerational relationship. On the other hand, Y6 expressed a different motivation that since his partner was active enough, he practiced on his own: “*My mother was fine and very active. So, I was not interested more to share my data with her. Instead, I tried to increase my step levels to have a fit body.*”

Finally, O4 and Y4 believed that if they were to be paired with a different partner to the one they had, they would have a better experience. O4: “*It would be better if I could check my daughter’s fitness data rather than that of my nephew. It doesn’t make much sense to me knowing his data.*” Her partner, the nephew, had similar comments about her. Y4: “*I wish I had a better relationship with my aunt, then I could communicate well with her using the tracker. If I could get paired with my mother, then I would definitely do more with the tracker.*” This finding shows the necessity of pairing partners with strong social bonds; those who have closer family ties. Otherwise, the lack of mutual interest might discourage intergenerational fitness practices.

### Barriers to engagement and user expectation

#### Instant awareness is more stimulating than weekly updates

All younger and older users reported checking their own step numbers significantly more often than their partners. While users checked their own data “several times per day”, they checked their partners’ data “several times per week”. Probing for the underlying reason for the decrease in the frequency of checking on their respective buddies, we found that users believed a lack of automatic sharing function reduced the potential opportunities to interact between users. O4: “*I could only see the total steps [of my partner] at the end of the week. I can never find out how many steps he has right away unless he shares it with me [via messaging]. It is not meaningful if we cannot see each other’s data right away.*” O5: “*To see the data both of us should post it. But she was busy and didn’t share enough.*” O6: “*It is difficult to compare myself with him on a daily basis. Once we started to use the tracker, we should see each other’s data even without sending it.*” We also received similar comments from younger users. Y3: “*The data could be shared automatically among partners.*” Y5: “*I wish to see how much my partner did at the end of the day.*”

#### Lack of playfulness in intergenerational app design

Although playfulness was an important practice during the individual use, older users did not comment on the playfulness later in the intergenerational settings. Whilst it is out of the scope of this study to evaluate the Fitbit app’s design, it appeared that the Community function lacks enough playfulness elements to engage intergenerational partners. One of the older users expressed his desire to have the tracker be more playful when using it together with his partner. O3: “*If I knew that I was going to win or lose the day, I could have approached it like a game. Currently, we can only share information, but we don’t act up it. I want to receive a notification of the winner of the day. I want to approach it more like a game.*”

#### Users need health literacy

Besides step and floor numbers, several older users (4/6) also reflected on other metrics of the tracker such as heart rate and sleep data. O1: “*I was also checking my heart rate data. When I took the floors up and down usually my heart rate goes up.*” O6: “*I had a little nap in the car. That [small data] was also displayed precisely. Look, you will find out I was taking a nap secretly (laughing).*” However, O4 believed only tracking heart rate and sleep is not informative enough: “*It is good to see my heart rate, but to be honest I don’t know what to do with that. I don’t have any merits in looking at these graphs. If my heart rate is high, is it good or bad? I need the tracker to interpret it for me.*” O1 reported giving her heart rate data to a 3rd party person to better understand her health condition: “*I reported my heart rate records to a health care provider in the fitness club.*”

#### Technology barriers

All older users (6/6) perceived technical barriers when interacting with the Community function. Four older users thought the design was not intuitive or easy to use. O1: “*I have a bad memory. Sometimes, I forgot where the button to send a message is. And then I tapped here and there, and I could send it. But next time I again forgot!*” O3: “*There were some technical terms/issues that I could not understand well, like how to use, how to add a friend, how to reply. Also, a lot of disconnections happened that I could not connect back.*” One of the younger users who said that her father faced many technical challenges expressed a need for easy to use technologies. Y2: “*It’s good if there’s something easy to use something that everyone can use and understand from elementary school student to older people.*”

## Discussion

We begin this section by exploring how our findings address the initial research questions. Later, we propose several design implications based on the insights obtained from our findings. Last, before closing the paper, we mention the limitations and future research directions.

### Revisiting the research questions

***RQ1. What are the intergenerational fitness tracking practices and what factors do facilitate or inhibit such practices?***

We found that comparability is a common practice for older adults to improve their motivation for physical activity. Our finding contrasts with prior studies that suggested people might respond poorly to within-family competitions (Grimes et al. 2009), or they might be not interested in comparing themselves with their friends due to differences in their physical capabilities (Gui et al. 2017). Nevertheless, it seems when it comes to familial intergenerational fitness practices, competition makes more sense, and in particular, older adults are enthusiastic in comparing themselves with their buddies.

We also identified the significant role of younger adults whose support was crucial for building a successful intergenerational experience. Younger partners, who were good matches to their older buddies, strongly supported this practice in different socio-technical aspects such as motivating the older partner, better understanding and caring for them and helping them overcome technology barriers. In particular, we found that “in-person” meetings and daily discourse are the main facilitators of intergeneration fitness practices.

On the other hand, some younger partners, who were not sufficiently interested in intergenerational relationships, failed to support their older partners. Moreover, despite intergenerational programs, technology barriers still remain.

***RQ2. How do these practices affect the social interaction of older adults with their younger family members?***

Intergenerational fitness practices seem to have increased social interactions between partners outside of dedicated physical exercise practice. We observed an increase in the number of family meetings.

These relationship practices may help older users use trackers better while improving their social bonds. The enhanced social interactions might occur because older users had many technical issues that they expect younger partners to resolve for them or simply because using fitness trackers together with family members creates new opportunities to bring people together “in-person”, “face-to-face”, specifically not remotely. The enhancement of social interactions may have further benefits, such as motivating older adults to participate in fitness activities.

### Implications for design and research

We draw twelve implications $(i_x)$ for the design and research of future fitness tracking devices. Table 3 summarizes these implications. We further discuss them in the following sections.

**Table 3:** Summary table of twelve implications

	Implication	Objective
$i_1$	Increasing the younger partners’ incentive	Enhancing partners’ engagement
$i_2$	Encouraging older adults to reverse interaction	Enhancing partners’ engagement
$i_3$	Giving equal weight to the requirements of both younger and older adults	Enhancing partners’ engagement
$i_4$	Using automatic and instant data-sharing features	Enhancing partners’ engagement
$i_5$	Considering privacy concerns of users when designing for $i_4$	Preserving peers’ privacy
$i_6$	Integrating intergenerational storytelling systems into fitness trackers	Facilitating daily discourse between peers
$i_7$	Using questionnaires before matching partners	Matching partners
$i_8$	Studying the effect of the relationship as a mediating variable	Matching partners
$i_9$	Using persuasive interventions to break the ice between partners	Matching partners
$i_{10}$	Employing social strategies such as competition and collaboration in the intergenerational context	Encouraging competition
$i_{11}$	Developing novel social features such as hybrid social engagement strategy	Encouraging competition
$i_{12}$	Using tutorials or virtual health agents to interpret health data	Facilitating the interpretation of health data

#### Increase partners’ willingness to engage

Older users tend to depend on or wait for younger counterparts to initiate interactions and validate their actions (cf. Sect. 5.2.2). It quickly became apparent that younger partners had a significant role in the success of intergenerational fitness practices (cf. Sect. 5.4). On the other hand, younger adults, compared with their older counterparts, usually have more responsibilities in daily life and are likely to be busy during the day. It is also possible that some older adults might still work. Even retired older adults might engage with other activities such as volunteer work. Therefore, both parties might have little choice to invest time in fitness tracking practices compared with their counterparts. Thus, we suggest:

$(i_1)$ Consider increasing the younger partners’ incentive to participate in the program, for example, using gamification techniques. Such incentives can be developed for instance by using the amount of interaction between partners as a metaphor for the health of older partners that being to inform younger partners that “the more you interact with your partners the more healthy they will be”. This could motivate younger partners to keep the interaction alive by regularly checking their partners’ data and frequently communicating with them, and in such a way to directly contribute to the well-being of their older partners; $(i_2)$ Consider designing features that encourage older adults to reverse interaction and support initiatives with their younger partners. For example, a fitness tracker could guide older partners to initiate the interaction by asking for assistance from their younger partners in daily physically-demanding tasks such as shopping or household chores; $(i_3)$ Given the younger partners’ significant role in the success of such programs, when designing an intergenerational fitness system, consider giving equal weight to the needs and preferences of both younger and older adults.

Because of busy lifestyles, younger users might forget to share their fitness information everyday. In such situations, older partners get frustrated because they cannot access information from their younger partners (cf. Sect. 5.5.1). On the other hand, for some younger users, it is important to monitor their older partners for the sake of health care (cf. Sect. 5.3.1, 2nd par.). Absence of remote monitoring can cause dramatic outcomes in critical moments and crisis, such as pandemics.^7^ This need is also confirmed by earlier studies, where different systems have been developed for remote health monitoring of older adults (Evans et al. 2016; Sasaki et al. 2007) and by recent fitness products (Ghosh et al. 2018).^8^ Thus, we suggest: $(i_4)$ Consider using automatic data-sharing features to visualize the physical activity of users instantly and continuously. See for example HealthyTogether (Chen and Pu 2014).

While our study itself did not examine the privacy concerns of partners in sharing fitness information, earlier studies (Vilaza and Bardram 2019; Li et al. 2020) noted that some users might be reluctant to share fitness information for reasons of privacy. Thus, we recommend: $(i_5)$ Consider privacy concerns of users when designing instant-sharing features. For instance, fitness systems should inform individuals about data being shared and the potential risks of sharing fitness information. See, for example, a work by Aktypi et al. (2017) that educates fitness tracker users by presenting potential privacy risks when sharing fitness data. Users should also be able to easily disable instant-sharing features (e.g., revoking access), when they feel their privacy could be violated.

#### Facilitate daily discourse and online communication between the generations

It was revealed that in-person communications between partners were an important part of intergenerational practices where partners could exchange their fitness activities face-to-face. Younger users were able to help their older partners use the tracker and the app in a proper way (cf. Sect. 5.3.2). We also found in-person meetings to be a meaningful opportunity to strengthen family bonds (cf. Sect. 5.3.1). However, such meetings usually occurred after the daytime when younger partners come back from work or when partners were on vacations. We observed that those users who were not able to meet every day due to distance or time constraints had less chance to get the full benefits of the program (cf. Sect. 5.3.2, last par.). A recent work (Welsh et al. 2018) designed a communication app to encourage conversation between young people and older people with dementia. In addition, other studies (Bentley et al. 2011; Jones and Ackerman 2018; Li et al. 2019) provided insights and design features for developing technologies for intergenerational story-telling practices. For example, Bentley et al. (2011) developed a location-based asynchronous communication app to stimulate intergenerational conversations. Thus, we recommend: $(i_6)$ Consider integrating the existing knowledge and guidelines for developing intergenerational communication and story-telling systems into fitness tracker design to leverage trackers’ capabilities and facilitate discourse between older and younger users. For instance, future design can consider logging meaningful interactions with the tracker [e.g., achieving a meaningful goal (Niess and Woundefinedniak 2018)] and making story-lines to support the story-telling propensity of older people in the fitness context.^9^

#### Find a well-matched partner for older users

Our findings highlighted a need to find compassionate younger family members to participate in intergenerational fitness practices (cf. Sect. 5.4). We observed different factors such as low confidence to engage in social activities, a busy lifestyle, and lack of emotional bonds between partners that detracted from intergenerational fitness practices. The positive role of intimacy between participants on social reinforcement was also argued by an earlier study (Lee and Lim 2015). However, despite these findings, further research is required to identify all major barriers. Verifying the motivation of younger users before starting the intergenerational practices is important, especially for the elderly partner. Thus, to build successful intergenerational experiences, we suggest: $(i_7)$ Before pairing intergenerational partners, consider using well-established selection criteria (e.g., questionnaires) or developing a questionnaire to measure the relationship (e.g., emotional bonds) between family members. It is also advisable to evaluate younger partners’ perceived social self-efficacy; $(i_8)$ Given that nature of the relationship between the relatives affects all phases of partnership’s practice, it would be useful to consider studying the effect of relationship as a mediating variable in future studies.

While our findings encourage pairing partners with close family ties, another stream of research should also focus on how family members with weaker bonds can be motivated to initiate their social interaction. An earlier study, proposed using “nudges” (Thaler and Sunstein 2008; Caraban et al. 2019) to improve social connectedness (Abouzied and Chen 2014) or enhance social sharing behavior (Huang et al. 2018). Such approaches could leverage the contribution of intergenerational fitness practices not only for family members with weaker bonds but also for strangers to build new intergenerational and inter-cultural relationships. Thus we suggest: $(i_9)$ Consider designing subtle persuasive interventions to break the ice between partners. For instance, consider designing nudges or using social norms such as reciprocity (i.e., feeling obligated to return a favor) (Cialdini 2009) in the fitness tracker design. Such interventions could also be useful for volunteer activities, for example, when a suitable relative is not available, and a stranger is assigned to an older person [see (Puussaar et al. 2017, p. 6938) for example].

#### Encourage competition

Comparability was one of the intergenerational fitness strategies that older users practiced (cf. Sect. 5.2.1). Most of the older users reported reflecting on their buddies’ fitness data, to compare it with their own data. This is in line with an earlier study (Dionigi et al. 2011) showing that older adults appreciate the challenges, such as competition. But at the same time, we found while participants during the individual use were engaged more with playfulness elements such as metaphors and badges, due to the app design, they did not have such opportunities later in the intergenerational settings (cf. Sect. 5.5.2). Gamification elements such as leaderboards have already been used in social fitness platforms (Gui et al. 2017). In addition, earlier studies (Chen and Pu 2014; Lin et al. 2006) have already investigated the dynamics of different social settings such as cooperation, competition, and collaboration in motivating users for physical activity. Nevertheless, these studies have not been tested with intergenerational users. Given these considerations, we suggest: $(i_{10})$ Consider studying social dynamics in *intergenerational* settings and employing novel social strategies in the tracker design to persuade users to compete or collaborate with each other.

In addition, our analysis showed older users approached their younger partner’s fitness data differently (cf. Sect. 5.2.2, 2nd par.). While they reported perceiving their own data as a contribution from daily exercises, they identified their younger partner’s data as a result of daily mobility due to work conditions. We believe this insight would lead to novel social design opportunities. Thus, we suggest: $(i_{11})$ Consider developing novel social features such as hybrid social engagement strategy (Chen and Pu 2014) where older users can change the weight factors of their own and their partner fitness data based on how they perceive their partner’s data. For example, an older partner who does jogs regularly might assign 80% coefficient for her or his own steps, and leave only 20% for the younger partner who walks for commuting.

#### Support for interpretation of health data

When we asked older participants “what kind of features they would like to see in the future tracker design”, all of them mentioned they expect their tracker to measure beyond-basic health features such as blood pressure (cf. Sect. 5.5.3). Older users also found it useful to track their sleep and heart rate data. However, despite the abundance of self-tracking mobile apps in the market, most of them lack enough sophisticated feedback to interpret health-related data. Our participants reported difficulties in interpreting such data and expressed a need to provide more knowledge about health data and how to respond to it. Indeed, this is consistent with an earlier study (Arcury et al. 2020) that older adults require technical support to improve their eHealth literacy. Also, younger partners were not able to provide such knowledge as it was out of their field of expertise. So, we suggest: $(i_{12})$ Consider providing tutorials or general diagnostics for both partners in the tracker app, informing how to interpret health records such as sleep and heart rate data, or consider designing a virtual health agent that can interpret the health data for older partners and their family.

## Limitations, future directions, and conclusion

### Limitations

The study is subject to limitations. First, we conducted a 4-week study. Although we found an enhanced social interactions between partners, this might occur because of the novelty effect, where both younger and older users were new to the trackers and they had enough stories to exchange. It is also possible that, our participants might be excited about the novel features of the intergenerational fitness tracker use, and later after the study, abandon the fitness tracker. However, it is worth mentioning that our main focus was not on habit changing, nor on the acceptance of technology. Rather, we probed elderlies’ perceptions and pain points while using intergenerational fitness trackers in “short-term”. Future studies could consider longer experiments to investigate habit formation and technology acceptance in intergenerational fitness practices. It would also be useful to consider a follow-up phase in future experiments to study the willingness of older adults to use the fitness tracker after completion of the study.

Second, as the warm-up period, all participants started the experiment using the trackers individually and then ended with the intergenerational settings. Although, the purpose of this study is not to make a comparison between individual and intergenerational settings, not counterbalancing the conditions could create an order effect. So, the effect of practices during the individual setting might carry over to the intergenerational ones. Nevertheless, counterbalancing the experimental conditions can also cause irreversibility. This means that if the intergenerational use was proposed before the individual one, withdrawing the social intervention in the middle of the experiment can negatively influence the results and the users behavior, and it may cause them to wonder why we would want them to diminish social interactions with their partners (this happened in our earlier lab experiments).

Third, we used snowball sampling for participant recruitment, where initial informants nominated other participants. This recruitment approach might cause sampling bias and limit the representativeness of our sample. Thus, the findings should be interpreted with caution.

### Future directions

Future research could also explore intergenerational fitness practices in different directions: (i) users with different cultural or socioeconomic backgrounds (e.g., users from western demographics) might experience intergenerational fitness practices differently and thus offer additional insights. Younger users may also have different attitudes toward intergenerational bonds and social interactions. Thus, future work can study intergenerational fitness practices in different contexts and demographics to provide further design implications; (ii) our work focused on the relationship between older adults and their adult children approaching their middle age. A recent study (Potapov and Marshall 2020) has discussed the co-design of a personal informatics app for youth. Other studies (Oygür et al. 2020, 2021; Saksono et al. 2020) investigated how children might use the fitness trackers and fitness apps together with their parents. But future work should also investigate how children in their adolescent years can participate in intergenerational fitness practice together with their grandparents; (iii) it would be also interesting if longitudinal studies (e.g., A/B testing) could compare an intergenerational group with an intragenerational old-old group to better understand the impact of the intergenerational settings on promoting physical activity.

### Conclusion

We carried out a qualitative study investigating digitally facilitated intergenerational fitness practices. We found the practices were effective in improving older adults’ social interactions with younger partners, thus strengthening family relationships. The practices significantly increased the awareness of the two generations, their respect for each other, their cares and interests, and it tended to promote new dimensions of mutual encouragement and support. We found that this kind of digitally facilitated program also promoted in-person meetings and daily discourses which became significant factors in helping older users to get support in overcoming technological barriers. Surprisingly, this relationship is reciprocal, although, in the beginning, the younger ones were more apt at using technology. The main pain points were also identified. The group dynamics and relatedness of the partners were crucial factors in making this particular program work. Through this in-depth qualitative study, our paper provides important foundations for understanding digital intergenerational fitness practices.

## Data Availability

Not applicable.

## Footnotes

1 https://www.extension.iastate.edu/life, last retrieved November 2021.

2 A fitness plug-in; built inside the WeChat platform.

3 https://www.latimes.com/business/story/2019-11-01/google-to-buy-fitness-wearables-giant-fitbit-for-about-2-1-billion, last retrieved November 2021.

4 The statistical information were collected from local media in Japanese.

5 An old-fashioned pedometer made in Japan. Manpo-Kei literally means 10,000 steps meter (Tudor-Locke 2003).

6 Given the qualitative nature of the study, the number of participants, and lack of significant difference, we cannot claim that intergenerational fitness practices improve physical activity.

7 For example, in the COVID-19 pandemic elderly people were found dead in a nursing home in Spain. See: https://www.bbc.com/news/world-europe-52014023, last retrieved November 2021.

8 See also Apple watchOS 7. It notifies family if it detects fall or irregular rhythm in older adults: https://www.apple.com/newsroom/2020/09/apple-extends-the-apple-watch-experience-to-the-entire-family/, last retrieved November 2021.

9 Note that an earlier work (Saksono and Parker 2017) studied story-telling between generations in the context of physical activity. But, this study did not consider older adults as one of the stakeholders of such practices.
